# Effect of Serendipity in an Encounter on Purchase Intention of Unexpected Products

**DOI:** 10.3389/fpsyg.2022.848907

**Published:** 2022-03-31

**Authors:** Shichang Liang, Yuxuan Chu, Min Zhang, Rulan Li, Bin Lan, Lingling He

**Affiliations:** ^1^College of Business Administration, Guangxi University, Nanning, China; ^2^College of Economics and Management, Nanning Normal University, Nanning, China

**Keywords:** serendipity, perceived risk (PR), unexpected products, purchase intention (PI), regulatory focus

## Abstract

Previous studies on the follow-up effect of serendipity mostly focused on the positive effects and less on the negative effects. Therefore, the purpose of this article is to investigate the negative effect of serendipity on the purchase intention of unexpected products. To verify all hypotheses in this article, we used online and offline survey data in China. Three experimental results showed that serendipity contains a certain degree of uncertainty, which will cause consumers’ perceived risk and decrease the purchase intention of unexpected products. Perceived risk plays a mediating role in the effect of serendipity on the purchase intention of unexpected products. Moreover, regulatory focus moderates the effect of serendipity on purchase intention of unexpected products. Specifically, for prevention-focused individuals, the negative effect of serendipity on the purchase intention of unexpected products is strengthened. For promotion-focused individuals, the negative effect of serendipity on the purchase intention of unexpected products is weakened. This article augments the understanding of the negative effects of serendipity and provides theoretical guidance and support for the management practice of marketers.

## Introduction

When serendipity in an encounter occurs, they could be full of uncertainty. Some people may be curious about the serendipity, whereas others may think that serendipity in an encounter has ruined their original purchase plan, resulting in hesitation. Serendipity refers to the discovery of valuable or pleasant things that are not sought for Aekyoung et al. (2021). Meanwhile, unexpected products refer to products that were found unexpectedly in the process of original purchase. Imagine such a shopping experience: The facial cleanser you often use has run out. At this time, you decide to go to the store and buy a new one. When you arrive at the store, you run into a random woman who has another brand of facial cleanser in her shopping cart, which catches your eye. Coincidentally, you just saw the advertisement for this facial cleanser on TV a few days ago. However, it is a product you unintentionally found and you haven’t used before. Therefore, you may perceive risk if you buy it because you may feel uncertain about it. In this scenario, do you prefer the planned product or the unexpected product? This is the question to be solved in this article.

The extant literature on serendipity covers topics such as consumers’ response to accidents (Heilman and Rao, 2002), the role of serendipity in promoting scientific and technological inventions (Murayama et al., 2015; Copeland, 2017; Arfini et al., 2020), serendipity in digital information environment (Lennart and Björneborn, 2016), serendipity in recommendation service (Kotkov et al., 2016), and discovering new opportunities in the field of entrepreneurship (Dew, 2009). Some scholars have studied the positive effect of serendipity on consumer behaviors. For example, Lindgreen and Vanhamme (2003) found that positive serendipity can bring positive value to consumers and generate positive emotions, thus promoting customer satisfaction. Dew (2009) proposed that serendipity can help entrepreneurs find new opportunities in the field of entrepreneurship because entrepreneurship is a series of random collisions. Brown (2005) showed that opportunities and serendipity played an important role in the historical development of Nike, Apple, P&G, and KFC. Aekyoung et al. (2021) showed that a high level of serendipity will enhance the sense of curiosity, and then affect a series of consumer consequences. However, these studies mainly focus on the positive effect of serendipity on consumer behaviors. Little is known about the negative effect of serendipity on consumer choice. Therefore, this article will address this gap by exploring the negative effect of serendipity in an encounter on consumer decision-making.

When serendipity occurs in an encounter, uncertain perception arises because serendipity contains randomness and opportunities (Friedel, 2001). Oglethorpe et al. (1987) found that a high level of uncertainty may lead to negative results, such as reducing the possibility of purchase. According to risk perception theory (Koay and Phau, 2018), when people perceive uncertainty, they will increase their risk perception. Therefore, this article proposes that serendipity will induce consumers’ perceived risk and then reduce the purchase intention of unexpected products. Moreover, according to regulatory focus theory (Higgins and Crowe, 1997; Friedel, 2001; Ku and Hung, 2018), promotion-focused people tend to achieve the positive results they want to pursue and are not afraid of risks, whereas prevention-focused people usually pay more attention to the worst result (Forster et al., 2003). Therefore, this article proposes that regulatory focus moderates the effect of serendipity on purchase intention of unexpected products. Specifically, for prevention-focused individuals, the negative effect of serendipity on the purchase intention of unexpected products is strengthened. For promotion-focused individuals, the negative effect of serendipity on the purchase intention of unexpected products is weakened.

This article presents three main theoretical contributions. First, this article explores the negative effect of serendipity on consumers’ decision-making. Previous studies focused on the positive effects of serendipity, whereas this article shows that serendipity in an encounter could lead to negative effects. Specifically, serendipity will induce consumers’ perceived risk and then reduce their purchase intention of unexpected products. This article addresses the gap of the negative effects of serendipity on consumer behaviors. Second, this article enriches the research on regulatory focus theory in the field of serendipity and found that the negative effect of serendipity on the purchase intention of unexpected products is strengthened only for prevention-focused individuals. Third, this article extends perceived risk as an intermediary variable to the field of serendipity and found that perceived risk plays an important role in the effect of serendipity on consumers’ decision-making.

## Background and Theoretical Development

### Serendipity

Serendipity is defined as the discovery of valuable or pleasant things that are not sought for Aekyoung et al. (2021). For example, when you are looking for something, you find something else interesting (Mirvahedi et al., 2017). Serendipity in the marketplace refers to a series of feelings generated in the purchase process, including consumers’ accidental discovery of products, services, or experiences on which they have no direct choice. This article defines serendipity as that person find something else similar but unexpected when they pursue what they want. Some scholars showed that the serendipitous experience is surprising and makes people feel lucky when the serendipity occurs in a positive, unexpected way that includes opportunities (Foster and Ford, 2003; Makri et al., 2014). The term “serendipity” originated in 1754. Horace Walpole described serendipity as an encounter wherein the title characters of the Persian fairy tale are making unexpected discoveries by accidents and sagacity, of things they were not in quest of Mcpherson and Cook (2001), which is described as a lucky discovery. Subsequently, serendipity has been used in different contexts, such as medical treatment (Ochsner, 1946), psychology (Williams et al., 1998), library stacks (Carr, 2015), information science (Kelloway et al., 2015), marketplace (Brown, 2005; Mirvahedi et al., 2017), and organizations (Cunha et al., 2010).

The present research focuses on the positive effect of serendipity. In the field of natural science, Friedel (2001) found that serendipity is an important factor to promote modern scientific and technological invention. For example, Newton discovered gravity under an apple tree and Archimedes accidentally discovered the way to calculate the volume of irregular objects (Stoskopf, 2005). Scholars hold the same opinion in the field of the marketplace. Lindgreen and Vanhamme (2003) proposed that positive serendipity has a positive effect on customer satisfaction. The more positive the serendipity is, the easier it is for consumers to gain satisfaction. Furthermore, in the consumption of recommendation services (such as music and film channels), when consumers are occasionally recommended a song they like, their satisfaction will be enhanced (Celma, 2010), because it will give consumers unexpected satisfaction. Kim and Tanford (2021) found that compared with functional products, hedonic products offer an unexpected discount, which will lead to consumers’ unplanned purchases. Gao and Mattila (2019) showed that for low-level consumers, taking an unexpected reward mode can produce higher donation intention. However, previous research mainly focuses on the positive effect of serendipity in an encounter, but less on the negative effects of serendipity. Uncertainty contained in the serendipity could bring a series of negative reactions for consumers who feel inconsistent with their expected goals, such as anger, disappointment, and anxiety (Goldstein et al., 2014). In addition, Aekyoung et al. (2021) showed that serendipity includes the feeling of surprise in some conditions, which leads to positive experience and enjoyment only by chance. For example, consumers see records that have been pursued for a long time appear on the shelves of retail stores. Serendipity does not always satisfy customers. Above all, scholars have not made a consistent conclusion on the positive and negative effects of serendipity, because they mainly focus on the positive effects of serendipity. Given that serendipity in an encounter is an accidental event (Green, 2004), uncertainty is involved in the process, and it often occurs as an unexpected event (Matt et al., 2014). Moreover, when the results of serendipity are inconsistent with their expectations, consumers will doubt this unexpected event. Therefore, this article explores the negative effect of serendipity in an encounter on consumer decision-making to address the gap in the literature on serendipity.

### Serendipity and Perceived Risk

Perceived risk is an important variable in the research on consumer behaviors. It refers to the uncertainty that consumers perceive in purchase decisions, including functional risk, time risk, privacy risk, and social risk (Yong et al., 2015). Previous research on perceived risk focuses on credibility and uncertainty. For example, Erdem and Swait (2004) explored the potential uncertainty of brand credibility, which affects consumers’ perceived risk. Chen and Yuan (2015) found that compared with booking hotels on transparent travel websites, consumers may feel higher uncertainty when booking on opaque travel websites, which will affect their perceived risk. Yu et al. (2018) found that for consumers who highly avoid uncertainty, adding quality labels to luxury products can help reduce their perceived risk. In addition, in measuring the risk involved in purchasing lottery tickets, Brachinger and Weber (1997) showed that no risk arises if uncertainty is not involved. Uncertainty is always an important factor affecting consumers’ perception of risk. Xiao et al. (2020) found that people are cautious in expressing a high level of hope when they perceive risk, because such expectation may lead to more negative behavioral intentions.

Serendipity is described as luck, opportunity, or destiny (Mirvahedi et al., 2017). However, people know that luck does not exist at any time, which often appears under certain circumstances (Friedel, 2001). For example, the probability that a person buys lottery tickets many times but never wins any prize is high. This process involves high uncertainty, thus, risks exist. Grange et al. (2018) found that consumers are more likely to find valuable products and gain purchase satisfaction when they search purposefully because they will carefully weigh the advantages and disadvantages of products. However, when they encounter unexpected products that are inconsistent with their expectations, consumers will be uncomfortable with the uncertainty Jahng and Jain (2000). Serendipity in an encounter will exacerbate the uncertainty in this condition. Thus, serendipity in an encounter disrupts consumers’ original plans and does not always satisfy consumers. Makri et al. (2014) also showed that serendipity involves unexpected situations and is inconsistent with their expectations. When serendipity is a negative experience, it will weaken consumers’ results, such as satisfaction and purchase intention (Aekyoung et al., 2021). Davison (2018) found that serendipity is not always under control, because it is an unexpected situation in the process of pursuing goals. Furthermore, research showed that serendipity is often beyond direct control, and consumers are likely to be affected because serendipity involves unexpected situations (Makri et al., 2014). For example, a person plans to go to the mall to buy a washing machine, but the merchant tells him (her) that he (she) is today’s lucky customer and plans to give him (her) a free washing machine. At this time, he (she) may have doubts about the free product. Bischof et al. (2020) found that consumers will perceive the inherent risk of providing unattractive products in subscription services when they accept surprise subscriptions because consumers do not follow their own planned choices, which will affect consumers’ choices and attitudes. Compared with the expected cash reward, consumers with internal participation will reduce the evaluation of focus brands when they receive unexpected cash rewards (Shibly and Chatterjee, 2020). These results showed that serendipity does not always have a positive effect on individuals because of its uncertainty, which will enhance individuals’ risk perception. Therefore, this article proposes that serendipity will induce consumers’ perceived risk.

### The Effect of Perceived Risk on Purchase Intention

Many studies have explored the effect of perceived risk on purchase intention. Lăzăroiu et al. (2020) found a negative effect of perceived risk on the purchase intention of consumers in social business platforms. Some scholars also explored the effect of perceived risk on purchase intention (Suki, 2007; Chen and Chang, 2012; Ecc and Yft, 2013; Yan et al., 2019). In addition, some scholars found that consumers’ risk perception hurts purchase intention of functional goods and hedonic goods (Chiu et al., 2014). Carina et al. (2021) showed that consumers’ purchase intention will be affected by perceived risk when they choose perfect (vs. imperfect) food. Negative behavioral consequences will be induced when consumers perceive risk, such as giving up buying and decreasing satisfaction (Lăzăroiu et al., 2020; Wang et al., 2022). Moreover, Yang et al. (2015) examined the effect of perceived risk and trust on consumers’ willingness to pay online. They found that low trust will enhance perceived risk and then reduce consumers’ willingness to pay online. Therefore, consumers will have low purchase intention when they perceive risk. This article proposes the following hypothesis.

*Hypothesis 1: Serendipity in an encounter will decrease consumers’ purchase intention of unexpected products.*

*Hypothesis 2: Perceived risk plays a mediating role in the effect of serendipity in an encounter on the purchase intention of unexpected products.*

### Moderating Effect of Regulatory Focus

According to regulatory focus theory, individuals can achieve their goals in two ways. One is by focusing on promotion, and the other is by focusing on prevention (Higgins and Tory, 1997; Pham and Avnet, 2004). The two approaches have differences in goal pursuit and behavior. Promotion-focused individuals have strong motivation to pursue goals, show enthusiasm, pay attention to growth, and show a positive attitude to results. By contrast, prevention-focused individuals pay attention to security, minimize negative results, and are eager to guarantee demand (Park et al., 2014). In addition, Forster et al. (2003) showed that promotion-focused people tend to adopt an exploratory information processing mode, emphasize speed, and strive to achieve positive results. However, prevention-focused people usually adopt a cautious information processing model, pay attention to the worst result, and strive to achieve results without a loss (Higgins and Crowe, 1997). Scholars found an interaction between regulatory focus and product type on purchase intention. Specifically, hedonic products can attract the attention of promotion-focused people, because they care about promoting the achievement of goals. Functional products can attract the attention of prevention-focused people because they care about whether the prevention goal can be achieved (Roy and Ng, 2012). In addition, Spanjol et al. (2011) examined how the regulatory focus motivation of individuals and leaders affects a team’s new product decision-making.

For prevention-focused individuals, negative results will be induced when they are faced with serendipity, which means a high level of uncertainty (Oglethorpe et al., 1987). Prevention-focused individuals tend to adopt conservative strategies in decision-making, thus ensuring their safety by avoiding losses (Molden and Finkel, 2010). Therefore, prevention-focused individuals are sensitive to risks, and their focus in the decision-making process is to avoid risks. In addition, scholars found that prevention-focused consumers worry about the performance risk of new products, thus reducing their purchase intention because new products have not been widely promoted and used in the market (Herzenstein et al., 2007). Hence, prevention-focused consumers show high-risk perception in the face of risk. Roberts and Mcbirnie (2008) showed that the external chain of events leading to unexpected discovery is not completely predicted or controlled by individuals, even if the serendipity could occur. As a result, prevention-focused individuals have a cautious response in the face of sudden and unpredictable situations, which enhance their perception of risk and reduce their purchase intention of unexpected products.

Promotion-focused individuals show positive behaviors when they are faced with serendipity. They are eager to pursue satisfactory results, prefer risks, and are willing to maximize their interests at the cost of heavy losses (Molden and Finkel, 2010). Promotion-focused people often seek risks, and they are eager for progress, achievement, and hope (Avnet and Higgins, 2006). Thus, when serendipity appears in an encounter, promotion-focused people are willing to reduce the uncertainty, and their perception of risk will be low. In addition, Campbell and Goodstein (2001) found that consumers are willing to choose unexpected products and prefer conflicting information when they perceived low risk. Compared with purchasing planned products, unexpected products are not within the scope of the plan. Promotion-focused individuals can quickly start the exploratory information processing mode when an unexpected condition occurs (Forster et al., 2003) and find valuable information among products (Wang et al., 2020). Therefore, when serendipity appears in an encounter, compared with prevention-focused individuals, promotion-focused individuals will perceive lower risk, which will weaken the negative effect of serendipity on the purchase intention of unexpected products.

*Hypothesis 3: Regulatory focus moderates the effect of serendipity on purchase intention of unexpected products.*

*Hypothesis 3a: For prevention-focused individuals, the negative effect of serendipity on the purchase intention of unexpected products is strengthened.*

*Hypothesis 3b: For promotion-focused individuals, the negative effect of serendipity on the purchase intention of unexpected products is weakened.*

**FIGURE 1:**
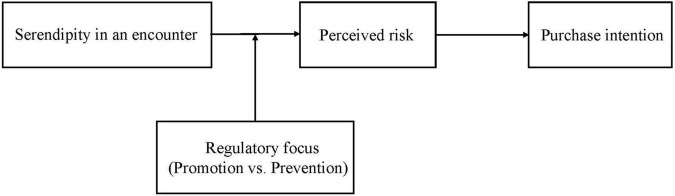
Theoretical model.

Figure 1 shows our theoretical model.

## Study 1

The purpose of study 1 is to test hypothesis 1, that is, serendipity in an encounter will decrease consumers’ purchase intention of unexpected products. We adopted the experimental manipulation method of Aekyoung et al. (2021) in study 1.

### Participants and Procedure

A total of 169 MBA post-graduates from a university in Southern China (56 males and 113 females, M age = 30.3 years) were recruited to participate in this study for course points as a reward. We adopt a single factor between-subjects design. First, participants were randomly divided into two groups. Participants of the serendipity group read the following situation: “Imagine that you often avail of the delivery service of W Express Company, where you have had a good consumption experience. One day, as usual, you were supposed to go to W Express Company to send a package. However, on the way to W Express Company, you unexpectedly found a new V Express Company. It is worth noting that this express company provides the same service as the express company you often go to.” Participants of the personal choice group read the following situation: “Imagine that you often avail of the delivery service of W Express Company, where you have had a good consumption experience. One day, as usual, you were supposed to go to W Express Company to send a package.” In addition, what needs to be noticed is that since it bears no serendipitous event at all, it is thus anticipated to find that the degree of surprise in the personal choice situation is to be significantly lower than that in the serendipity situation. We will verify this finding in the next manipulation check.

### Measures

#### Feelings of Serendipity

Participants were asked to answer questions regarding their feelings of serendipity. Three items developed by Aekyoung et al. (2021) were used: “I feel that the express company I just saw was a good surprise in the process of choosing an express company;” “It’s a great surprise to see this express company in the process of choosing an express company;” “I feel that the express company I saw was an unexpected discovery in the process of choosing an express company.” Participants rated their answers on a seven-point Likert scale (1 = “strongly disagree,” 7 = “strongly agree”).

#### Purchase Intention

Participants were then asked to fill in the scale of purchase intention developed by Lan et al. (2021). The items were: “I will choose to send express in the express company;” “I will recommend the express company to others;” “Next time I send express, I will choose the express company first.”

#### Brand Familiarity

We asked the participants about their familiarity with the brand. An item included: “I am very familiar with this brand.” After the experiment, each participant was given corresponding course points as a reward.

### Results

#### Manipulation Check

According to the independent sample *t*-test analysis, the participants thought that the degree of surprise in the serendipity situation (*M* = 5.62) was significantly higher than that in the personal choice situation [*M* = 3.13; *t*_(167)_ = 15.57, *p* < 0.001]. This result shows that the manipulation of serendipity is successful.

#### The Effect of Serendipity on the Purchase Intention of Unexpected Products

Participants’ purchase intention is significantly different in the two situations of serendipity and personal choice. The score of subjects in the serendipity situation (*M* = 5.35) is significantly lower than that in the personal choice situation [*M* = 6.03; *t*_(167)_ = 5.13, *p* < 0.05]. In addition, the results showed that brand familiarity is not an driver of our effect [*F*_(1, 167)_ = 0.002, *p* = 0.965].

### Discussion

The results of study 1 show that when serendipity in an encounter happens, consumers’ purchase intention will decrease. Thus, hypothesis 1 is supported. However, the stimuli of serendipity and personal choice group in study 1 have two different brands; hence, the experimental results may be affected by brand differences. To exclude the potential interference caused by brand factors, we carried out study 2.

## Study 2

On the one hand, the purpose of study 2 is to verify the mediating role of perceived risk in the effect of serendipity on the purchase intention of unexpected products (H2). On the other hand, we want to exclude the influence of competing brands in the stimuli used in study 1. Potential interference may affect the experimental results because of different brands. In addition, we designed an experiment scenario in which participants imagine themselves going to the shopping mall. Serendipity may occur in shopping malls.

### Participants and Procedure

A total of 165 participants from a community in Southern China (55 males and 110 females, M age = 27.9 years) were recruited to participate in the experiment. One participant who failed the attention test was excluded; hence, 164 participants were retained. A single factor between-subjects design was adopted. Participants were randomly divided into two groups. The participants of the serendipity group read the following situation: “Imagine that you love Leshi’s potato chips of cucumber flavor. At this time, you go to a nearby shopping mall to buy some. However, you are surprised to find that Leshi has launched another potato chips of sour cucumber flavor on the shelf, which has a similar but unique taste to your cucumber flavor.” The participants of the personal choice group read the following situation: “Imagine that you love Leshi’s potato chips of cucumber flavor. At this time, you go to a nearby shopping mall to buy some. You find the one you love on the shelf.” Meanwhile, the inference of the degree of surprise in the two groups is similar to the preliminary inference in study 1.

### Measures

#### Feelings of Serendipity

Participants were asked to answer questions regarding their feelings of serendipity. We used the scale by Aekyoung et al. (2021), which included items such as “I feel that the potato chip I just saw was a good surprise for me in the process of choosing a potato chip;” “It’s a great surprise to see this potato chip in the process of choosing a potato chip;” “I feel that the potato chip I saw was an unexpected discovery in the process of choosing a potato chip.” Participants rated their answers on a seven-point Likert scale (1 = “strongly disagree,” 7 = “strongly agree”).

#### Perceived Risk

Participants were then asked to answer the items on perceived risk developed by Séquier et al. (2002). Some examples are “I’m worried that the product is inconsistent with the description;” “I’m worried that a lot of trouble will come up in the after-sales service of the product; and “I still have a lot of questions about the quality of the product.”

#### Purchase Intention

Participants were asked to fill in the scale of purchase intention used by Lan et al. (2021), specifically, “If necessary, I will choose to buy the product;” “I will recommend the product to others;” “Next time I buy things, I will choose the product first.”

#### Expectations

In addition, we measured the variable of expectation as alternative explanations, specifically, “how high were your expectations about the product before you got it?” After the experiment, each participant was given gift rewards.

### Results

#### Manipulation Check

According to the independent sample *t*-test analysis, the participants thought that the degree of surprise in the serendipity situation (*M* = 5.71) was significantly higher than that in the personal choice situation [*M* = 3.37; *t*_(162)_ = 15.68, *p* < 0.001]. This result shows that the manipulation of serendipity is successful. In addition, no significant difference was found in expectations [*F*_(1, 162)_ = 0.05, *p* = 0.821], showing that the findings cannot be explained by expectations.

#### The Effect of Serendipity on the Purchase Intention of Unexpected Products

Serendipity in an encounter has a negative effect on purchase intention of unexpected products [M serendipity = 5.39, *SD* = 0.82, M personal choice = 5.74, *SD* = 0.90, *F*_(1, 162)_ = 6.93, *p* < 0.01]. Therefore, hypothesis 1 is supported.

#### Mediation Analysis of Perceived Risk

**FIGURE 2:**
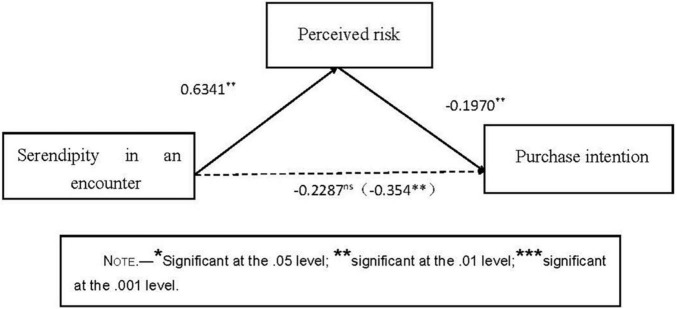
Mediation analysis of perceived risk.

We used bootstrapping analysis (Process model 4; Hayes, 2017) to test the mediating effect of perceived risk. In the model with perceived risk as to the dependent variable, serendipity affects perceived risk (β = 0.6341, *p* < 0.01). In the model with purchase intention as the dependent variable, perceived risk significantly affects purchase intention (β = −0.1970, *p* < 0.01). The mediating path of the influence of serendipity in an encounter on purchase intention is significant (indirect effect = −0.1249, *SE* = 0.0546, 95% CI: [−0.2482, −0.0336]). After the intermediary variable is controlled, the direct effect becomes non-significant (direct effect = -0.2287, *SE* = 0.1328, 95% CI: [−0.4909, 0.0335]). Figure 2 shows the specific path coefficient. The results showed that the mediating effect of perceived risk is significant.

### Discussion

The experimental results show that when the potential influence caused by brand factors is controlled, serendipity in an encounter will still affect participants’ purchase intention of unexpected products. This result further supports hypothesis 1. In addition, study 2 verifies that perceived risk plays an intermediary role in the effect of serendipity on the purchase intention of unexpected products.

## Study 3

Study 3 examined whether regulatory focus plays a moderate role in the effect of serendipity on the purchase intention of unexpected products. We proposed that when promotion-focused consumers have positive and active behavior, serendipity will weaken the negative effect of consumers on the purchase intention of unexpected products. To test this hypothesis, we carried out study 3. We used the manipulation method of serendipity by Aekyoung et al. (2021). For the manipulation method of regulatory focus, we refer to the research of Baas et al. (2011).

### Participants and Procedure

We recruited 242 participants (93 males and 149 females, M age = 30.3 years) on the Credamo platform that was similar to MTurk and founded by the Chinese. First, participants were randomly divided into two groups. All participants are told to complete two unrelated tasks. In the first task, they needed to complete the startup task of regulatory focus. Promotion-focused participants were asked to write an experience in which they achieved a positive result, whereas prevention-focused participants were asked to write an experience in which they avoided a negative result. In addition, they were required to describe the experience in concrete and vivid language, so that people can imagine the situation according to their description. After completing the task, they were told that they would continue with the second task. In the second task, participants were required to read the following scenarios. “Imagine that you are going to subscribe to a novel. You decide to go to a bookstore to buy the subscription.” Each participant of the serendipity group was randomly assigned by the bookstore owner to one of six novels according to the public’s preferences. Participants of the personal choice group chose one of the six novels on their own. Then, the participants were asked to answer some questions.

### Measures

#### Regulatory Focus

Participants completed a three-item scale of regulatory focus developed by Roy and Ng (2012). The items are: “do you prefer to do the right thing or what you want to do;” “do you prefer to avoid risks or to seek risks;” “do you prefer to avoid problems or solve problems?” Participants rated their answers on a seven-point Likert scale (1 = “strongly disagree,” 7 = “strongly agree”).

#### Feelings of Serendipity

Participants were asked to answer questions regarding their feelings of serendipity, specifically, “The novel I just saw was a good surprise for me in the process of selecting a novel;” “Considering the novel selection process, it’s a great surprise to see this novel;” “The novel was an unexpected discovery in the process of selecting a novel.”

#### Purchase Intention

Participants were then asked to fill in the scale of purchase intention developed by Lan et al. (2021), specifically, “if necessary, I will choose to buy the novel;” “I will recommend the novel to others;” “Next time I buy things, I will choose the novel first.”

#### Curiosity

We measured curiosity to exclude the alternative explanation, specifically, “how high was your curiosity about the product before you got it?” After the experiment, each participant was given gift rewards.

### Results

#### Manipulation Check

According to the independent sample *t*-test analysis, taking the degree of surprise as the dependent variable, the score of participants in the serendipity group (*M* = 5.23) is significantly higher than that in the personal choice group [*M* = 4.66; *t*_(240)_ = 4.05, *p* < 0.01]. Therefore, the manipulation of serendipity is successful. In addition, the results of one-way ANOVA showed that compared with participants in the prevention-focus group (*M* = 3.16, *SD* = 0.95), participants in the promotion-focus group were less afraid of risk [*M* = 5.21, *SD* = 0.92, *F*_(1, 240)_ = 290.53, *p* < 0.001]. Therefore, the regulatory focus manipulation is successful.

#### Purchase Intention

**FIGURE 3:**
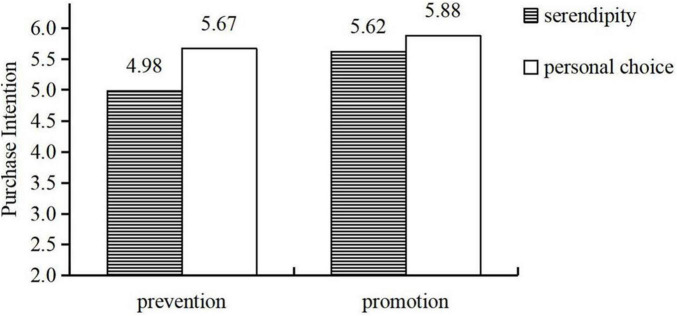
The moderate effect of regulatory focus.

2 × 2 ANOVA on purchase intention was used to test the interactive effect. The results showed that the main effect of serendipity is significant [*F*_(1, 240)_ = 24.74, *p* < 0.001], and the interaction was also significant [*F*_(1, 240)_ = 5.13, *p* < 0.05]. For prevention-focused individuals, compared with participants in the personal choice group, serendipity decreased their purchase intention of unexpected products [M personal choice = 5.67, *SD* = 0.78 vs. M serendipity = 4.98, *SD* = 0.80; *F*_(1, 240)_ = 22.77, *p* < 0.001]. For promotion-focused individuals, the negative effect of serendipity on the purchase intention of unexpected products was weakened [M personal choice = 5.88, *SD* = 0.67 vs. M serendipity = 5.62, *SD* = 0.67; *F*_(1, 240)_ = 4.35, *p* < 0.05] (see Figure 3). In addition, the results showed that curiosity is not an driver of our effect [*F*_*(1, 240)*_ = 0.449, *p* = 0.503]. The results of three studies are summarized (see Table 1).

**TABLE 1:** Summary of results of by study condition.

Study 1				
**Express service scenario; *N* = 169, 56 males, M_age_ = 30.3 years, MBA postgraduates**		

	**Serendipity**	**Personal choice**

Feelings of serendipity	5.62	3.13
Purchase intention	5.35	6.03
Main Finding: When serendipity in an encounter happens, consumers’ purchase intention will decrease.				

**Study 2**				

**Potato chip scenario; N = 164, 55 males, M_age_ = 27.9 years, Community**				

	**Serendipity**	**Personal choice**

Feelings of serendipity	5.71	3.37
Purchase intention	5.39	5.74
Perceived risk	Indirect effect = –0.1249, SE = 0.0546, 95% CI: [–0.2482, –0.0336] direct effect = –0.2287, SE = 0.1328, 95% CI: [–0.4909, 0.0335]	
Main Finding: When the potential influence caused by brand factors is controlled, serendipity in an encounter will still affect participants’ purchase intention of unexpected products.				


**Study 3**				

**Bookstore scenario; *N* = 242, 93 males, M_age_ = 30.3 years, Credamo platform**				

	**Serendipity**		**Personal Choice**	
**Regulatory Focus Group**	**Promotion**	**Prevention**	**Promotion**	**Prevention**

Purchase intention	5.62	4.98	5.88	5.67
Regulatory focus	5.23	3.32	5.19	3.01
Feelings of serendipity	5.47	5.00	4.89	4.44
Main Finding: Serendipity has a negative effect on consumers’ purchase intention of unexpected products. In addition, regulatory focus moderates the negative effect of serendipity on the purchase intention of unexpected products.				

### Discussion

The above experimental results showed that serendipity hurts consumers’ purchase intention of unexpected products. Specifically, owing to high uncertainty, consumers will perceive high risks when serendipity in an encounter appears, which hurts the purchase intention of unexpected products (H1). In addition, regulatory focus moderates the negative effect of serendipity on the purchase intention of unexpected products. Specifically, for prevention-focused individuals, the negative effect of serendipity on the purchase intention of unexpected products is strengthened. For promotion-focused individuals, the negative effect of serendipity on the purchase intention of unexpected products is weakened. H3 is supported.

## General Discussion

Serendipity plays an important role in consumers’ decision-making. According to regulatory focus theory, this article examines the interactive effect of serendipity and regulatory focus on the purchase intention of unexpected products. Moreover, perceived risk plays a mediating role in the relationship. This article verifies the hypotheses through three experiments. Study 1 used different express companies as stimuli to verify the negative effect of serendipity on the purchase intention of unexpected products (H1). To exclude the potential interference caused by different brand factors on the experimental results, study 2 was conducted to verify the main effect by using two different variants of the same brand. Additionally, study 2 verified the intermediary role of perceived risk in the negative effect of serendipity on the purchase intention of unexpected products (H2). Furthermore, study 3 verified the boundary conditions of the effect of serendipity on purchase intention (H3). The results showed that serendipity has significantly different effects on the purchase intention of unexpected products among consumers with different target motives. When serendipity in an encounter appears, the purchase intention of unexpected products will be lowered significantly in prevention-focused individuals compared with promotion-focused individuals.

### Theoretical Implications

First, this article enriches the literature on serendipity. Previous studies mainly focused on the positive effects of serendipity, and less on the negative effects of serendipity. Our research reveals that consumers induce less purchase intention of unexpected products when they experience serendipity than when they experience personal choice. The extant literature on serendipity showed that serendipity in an encounter can bri many benefits from different fields and perspectives, such as the exploration of business opportunities on the platform for technology companies (Moretto and Vasilchenko, 2011), the source of creative story ideas for media reporters (Bird-Meyer et al., 2019), the possibility of information seeking behaviors for lawyers (Solomon and Bronstein, 2016). However, these studies mainly focus on the positive effect of serendipity. Serendipity involves uncertainty, which entails fortuity. Evidence shows that serendipity can be positive or negative (Loewenstein, 1994; Calvo and Castillo, 2001). Therefore, this article enriches the research on serendipity by examining the negative effects of serendipity in an encounter.

Second, this article extends the research of regulatory focus theory to the field of serendipity. Promotion-focused individuals and prevention-focused individuals have different behavioral motivations. Previous studies on these differences are mainly reflected in time (Cassie et al., 2008) and information processing (Yoon et al., 2012; Dong et al., 2018), but few studies focused on the effect of different target motivations on serendipity. In this article, when serendipity in an encounter appears, prevention-focused individuals will have lower purchase intention of unexpected products compared with promotion-focused individuals. By contrast, for promotion-focused individuals, the negative effect of serendipity on the purchase intention of unexpected products is weakened.

Finally, this article expands the theoretical research of perceived risk in the field of serendipity. Previous studies on perceived risk mainly divided risk into functional risk, time risk, privacy risk, and social risk (Yong et al., 2015). This article holds that serendipity is uncertain and difficult to control. Unlike planned products, serendipity will increase consumers’ perceived risk of unexpected products. Therefore, this article enriches the research on perceived risk.

### Practical Implications

These findings provide important managerial implications for retailers and consumers in marketing activities.

First, our findings showed that serendipity in an encounter will decrease consumers’ purchase intention of unexpected products. Therefore, retailers can make different marketing strategies based on our recommendations. On the one hand, marketers should avoid serendipity to reduce regular customers’ perception of risk in the process of product promotion. On the other hand, marketers could provide new offerings to new consumers to help them explore interesting and enjoyable commodities in serendipitous encounters.

Second, our findings revealed that perceived risk plays an intermediary role in serendipity on consumers’ purchase intention of unexpected products. Retailers should consider enhancing consumer experiences by reducing their uncertainty perception of products. Therefore, various advantages of products could be specified in detail to reduce consumers’ risk perception, thereby improving sales in the product promotion activities.

Third, marketers should formulate corresponding product promotion strategies according to consumers with different motives. The negative effect of serendipity on consumers’ purchase intention of unexpected products varies between promotion-focused and prevention-focused individuals. For prevention-focused consumers, retailers should try to avoid making consumers perceive that the goods are serendipity. This strategy may be effective. For promotion-focused consumers, retailers could create surprising events and provide new products, which can stimulate consumers’ desire for curiosity. Therefore, businesses should arrange their marketing activities according to different consumer groups.

### Limitations and Future Research

This article has limitations. First, this article did not take into account cultural differences (e.g., Eastern vs. Western) in the influence of serendipity on the purchase intention of unexpected products. Individual cognition of serendipity varies due to Eastern-Western cultural differences (Karimova et al., 2020), which may lead to different research conclusions. Second, this article mainly studies the internal mechanism of perceived risk as to the intermediary variable in the effect of serendipity on purchase intention. Scholars have explored variables such as feelings of serendipity (Aekyoung et al., 2021). Other variables can be introduced in the future to explore the effect of serendipity on consumer behaviors. Third, this article only focuses on the boundary condition related to individuals. However, the effect of serendipity on purchase intention may also be affected by other factors such as product type and brand awareness. Therefore, future research is needed to examine whether brand awareness and product type al the serendipity effect.

## Data Availability Statement

The original contributions presented in the study are included in the article/Supplementary Material, further inquiries can be directed to the corresponding author/s.

## Ethics Statement

Ethical review and approval was not required for the study on human participants in accordance with the local legislation and institutional requirements. The patients/participants provided their written informed consent to participate in this study.

## Author Contributions

SL, MZ, and YC contributed to the conception of the manuscript and helped to perform the analysis with constructive discussions. SL and MZ performed the experiment. MZ and RL contributed significantly to analysis and manuscript preparation. BL and LH performed the data analyses and wrote the manuscript. All authors contributed to the article and approved the submitted version.

## Conflict of Interest

The authors declare that the research was conducted in the absence of any commercial or financial relationships that could be construed as a potential conflict of interest.

## Publisher’s Note

All claims expressed in this article are solely those of the authors and do not necessarily represent those of their affiliated organizations, or those of the publisher, the editors and the reviewers. Any product that may be evaluated in this article, or claim that may be made by its manufacturer, is not guaranteed or endorsed by the publisher.
